# Access to childhood cancer medicines in South Africa: a health systems analysis of barriers and enablers

**DOI:** 10.1080/20523211.2024.2372033

**Published:** 2024-07-12

**Authors:** Iris R. Joosse, Hendrika A. van den Ham, Aukje K. Mantel-Teeuwisse, Velisha A. Perumal-Pillay, Fatima Suleman

**Affiliations:** a Utrecht WHO Collaborating Centre for Pharmaceutical Policy and Regulation, Division of Pharmacoepidemiology and Clinical Pharmacology, Utrecht Institute for Pharmaceutical Sciences (UIPS), Utrecht University, Utrecht, The Netherlands; b WHO Collaborating Centre for Pharmaceutical Policy and Evidence Based Practice, School of Health Sciences, University of KwaZulu-Natal, Durban, South Africa

**Keywords:** South Africa, childhood cancer, access to medicines, health systems analysis, qualitative research

## Abstract

**Background:**

We sought to identify what barriers and facilitators determine current perceived access to childhood cancer care in South Africa through in-depth interviews with stakeholders in South Africa’s public and private sectors.

**Methods:**

Qualitative semi-structured interviews were conducted with 29 key health system stakeholders, including policy-makers and regulators, medical insurance scheme informants, medicine suppliers, healthcare providers and civil society stakeholders. Identified barriers and facilitators in access to medicines and broader care were structured according to the pharmaceutical value chain (PVC).

**Results:**

Barriers and facilitators were identified across all components of the PVC. Key barriers included (1) a lack of political commitment to childhood cancers, (2) discontinuation of essential chemotherapeutics, (3) incomplete insurance coverage for childhood cancers, (4) stock-outs of essential medicines, (5) the inability to access care, including travel to healthcare facilities and (6) low awareness on childhood cancers among primary healthcare (PHC) workers. Proposed priority interventions included pricing flexibilities, increased transparency and consistency in decision-making and healthcare spending, and improved training of PHC staff, nurses and pharmacists on childhood cancers.

**Conclusion:**

This first comprehensive study of determinants of access to medicines used in childhood cancer in South Africa provides context-specific evidence for targeted policy development.

## Introduction

Medicines are a core modality of childhood cancer treatment and are vital for survival and quality of life. Improving and sustaining access to essential oncology medicines is crucial for countries aiming to reduce cancer mortality and associated disease burden among their children (WHO, 2021). South Africa is among those low- and middle-income countries (LMIC) wanting to reach the World Health Organisation’s (WHO) Global Initiative for Childhood Cancer (GICC) target of at least 60% overall survival for children (WHO, 2021), with current national survival rates of about 50% (Stones et al., 2014; Ward et al., 2019). Barriers in access to cancer medicines for the general population – including unaffordable medicines, stock-outs and inconsistent drug supplies and discontinued manufacturing of medicines by industry (Mattila et al., 2021; Meyer et al., 2021; Modisakeng et al., 2020) – may also affect children. Besides a lack of access, other factors that are reported to contribute to poorer survival rates in this group are delays in diagnosis resulting in advanced disease and a worse prognosis, lack of treatment capacity, physical barriers to access care services and treatment abandonment (Stefan & Siemonsma, 2011).

South Africa currently has a two-tiered healthcare system (Michel et al., 2020; Suleman & Gray, 2017). The proposed National Health Insurance (NHI) – with a centralised health financing scheme – is intended to significantly reduce the pervasive health inequities experienced by the socio-economically disadvantaged (Gordon et al., 2020). Until NHI has been achieved, healthcare is offered to all South Africans for a small fee relative to their income in the public – government-funded – sector. Some groups, such as children under 6 years of age and the poorest, are exempted from these fees. Approximately 84% of South Africans depend on the public sector for their healthcare (Naidoo, 2012), which is further characterised by a complex pharmaceutical system, in which core processes such as medicine registration and the selection of essential medicines are managed by national bodies, whereas subsequent medicine procurement and care provision are organised provincially. Patients at private sector hospitals and clinics pay for healthcare via medical aid schemes (i.e. insurance) or are faced with out-of-pocket (OOP) payments (Mattila et al., 2021). In 2020, only about 15% of the population belonged to a medical scheme (Council for Medical Schemes, 2020).

To reduce South Africa’s childhood cancer mortality and health inequities, a better understanding of how the various pharmaceutical processes may contribute to the inaccessibility of cancer medicines is needed. The present study aimed to conduct a health systems analysis of barriers and enablers in accessing medicines used in paediatric oncology – including both antineoplastics and supportive care medicines (i.e. anti-emetics, analgesics, palliative care medicines, etc.) – in South Africa. This study can aid in strengthening the health system and identifying crucial policy development areas as South Africa moves towards implementing NHI.

## Methods

### Participants

Invitations to participate in this study were sent via email to 57 stakeholders in South Africa’s pharmaceutical value chain. Five key stakeholder groups: (1) policy-makers and regulators, (2) medical insurance scheme representatives, (3) medicine suppliers, (4) healthcare providers and (5) civil society stakeholders were chosen to represent all steps in the pharmaceutical value chain (i.e. policy and legislation, medicine regulation, financing and pricing, selection, reimbursement, supply and procurement, healthcare delivery, dispensing, use, monitoring and surveillance). Participants were initially identified through the professional network of FS and purposefully selected for their involvement with paediatric oncology medicines, but their activities did not need to be confined to childhood cancers only. Those exclusively involved in adult oncology were excluded from the study. The recruitment process was further informed by referrals from participants during the interview process.

### Interview guide

We developed a semi-structured qualitative interview guide drawing from the Paediatric Oncology Systematic Integration Tool (POSIT) to understand key aspects that influence access to childhood cancer medicines (Maser et al., 2020). Four main categories used in the interview guide mirrored those of POSIT: *governance*, *financing*, *social aspects* and *medicine delivery* (adapted from *service delivery*). Within these broad categories, open-ended interview questions were constructed to explore barriers and facilitators that stakeholders experienced or perceived in accessing childhood cancer medicines in both the public and the private healthcare sectors (see Supplemental Material Appendix 1). A draft of the interview guide was tested in a mock interview and piloted with one participant, which led to minor refinements of the guide.

### Data collection and analysis

All interviews were conducted in English from September to November 2022 by IRJ, an academic researcher with prior experience in conducting interviews and who had no previous connection with the participants. Interviews were conducted online or in a convenient location close to the participants’ place of residence or work and lasted approximately 45 min. All interviews were audiotaped after written and verbal consent from the participant and notes were made during the interviews. Following verbatim transcription, two transcripts were coded together by IRJ and FS to develop a robust coding approach and ensure consistent interpretation. The remaining transcripts were coded by the first author alone. As validation, the coding of five interviews (one per stakeholder group) was verified by researcher HAvdH to ensure that no themes were missed and themes were coded consistently. Discussions were held to clarify any disagreements in coding and to reach a consensus. Subsequent thematic analysis took place through a mixed approach. The deductive analysis was based on components of the health system in which the pharmaceutical chain resides, which includes two national contextual components (policy and legislation; monitoring and surveillance) and eight functional components of the pharmaceutical value chain (medicine regulation; financing and pricing; selection; reimbursement; supply and procurement; healthcare delivery; dispensing; use) (Joosse et al., 2024). The inductive analysis followed a modified grounded theory approach (Charmaz, 2014) where data was coded iteratively to capture emergent themes. Confidentiality of participants was maintained, and data were stored in line with legal requirements such as the Protection of Personal Information Act (POPIA).

## Results

**Figure 1.**
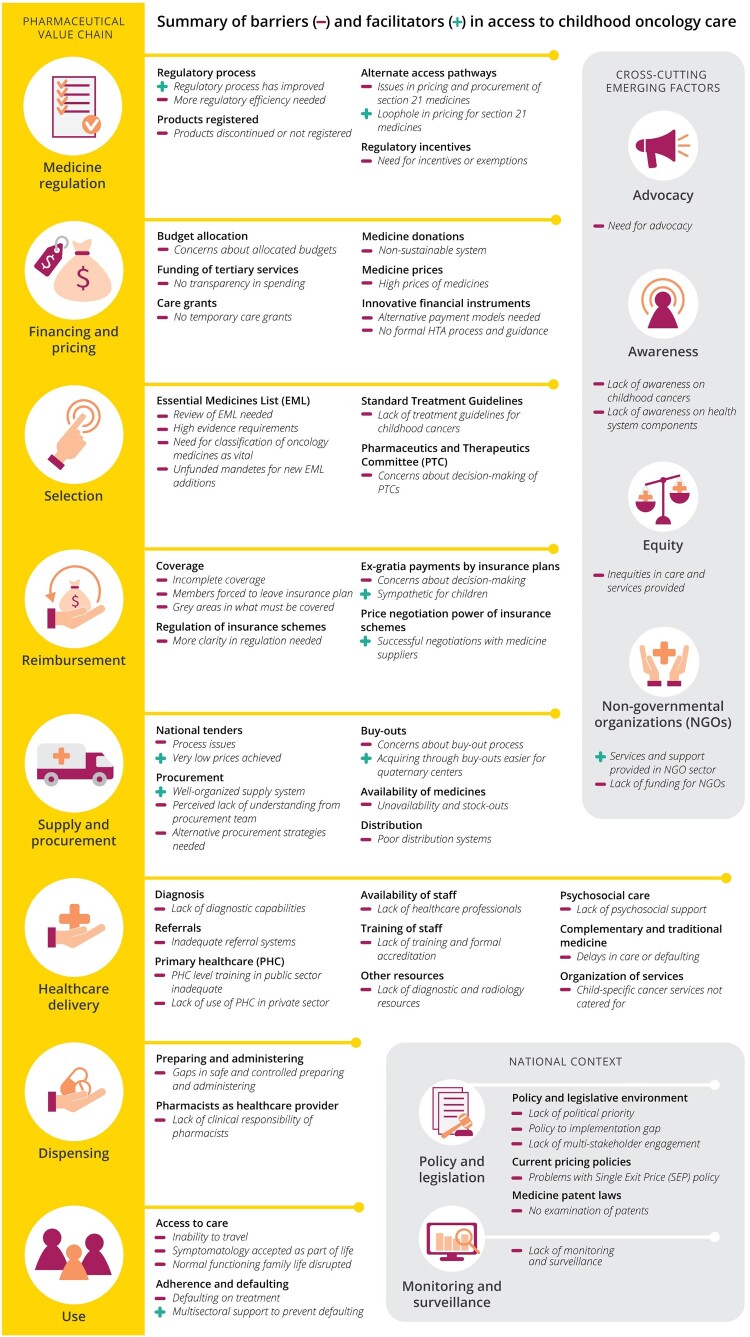
Overview of identified barriers and facilitators in access to paediatric oncology medicines and care.

A total of 29 stakeholders responded positively to our invitation and participated in qualitative in-depth interviews (7 policy-makers and regulators, 5 medical insurance scheme representatives, 7 medicine suppliers, 6 healthcare providers and 4 civil society stakeholders). Reasons for not wanting to participate included not considering themselves an expert in (childhood) oncology, or having left the field. An overview of participant characteristics is provided in Supplemental Material Appendix 2. An overview of identified barriers and facilitators, structured according to the pharmaceutical value chain, is provided in Figure 1. In addition to these, four cross-cutting themes emerged during data analysis, which intersect multiple components (advocacy; awareness; equity; non-governmental organisations (NGOs)). Additional stakeholder quotes are presented in Supplemental Material Appendix 3.

### Policy and legislation

Participants repeatedly indicated that paediatric cancers are not a priority due to the small number of children affected compared to adult cancers (and other disease areas). Having no official definition for what constitutes a rare disease complicates this political prioritisation. In formulating pharmaceutical policies, both policy-makers and medicine suppliers indicated a lack of constructive dialogue between the government and affected stakeholders. It was also indicated that – although a ministerial advisory committee and national cancer strategic framework (NCSF) for cancers exists – it does not include a clear policy for paediatric oncology specifically. Additionally, clarity on its operationalisation and subsequent implementation is wanting, likely undermined by a lack of capacity at the government level. To ensure the implementation of drafted policies, the alignment of policy development and funding through treasury needs to be re-examined.

Prices of medicines in the private sector are currently regulated through the Single Exit Price (SEP) policy, which dictates a universal price at which medicines are sold by the manufacturer to all distributors/dispensers in the country, also removing previously allowed discounts. The manufacturer is free to set the SEP, which must be disclosed publicly. Participants widely agreed that although the SEP policy increased accountability when first implemented in 2004, it now induces higher pricing because suppliers fear other countries use the disclosed prices for international reference pricing. The policy was not considered transparent by most participants, as manufacturers are still free to determine their prices. The lack of flexibility in pricing complicates reasonable pricing or discounting for rare diseases such as paediatric cancers. Finally, South African patent laws need to be revisited, as medicine patents are not sufficiently examined before granting them, thereby also contributing to higher pricing.

In the single exit pricing system, there is, as far as I’m concerned, no transparency, even though government sees it as a transparent system. What is transparent is the price. Yes, we advertise the price. But it’s still the pharmaceutical company that is actually deciding and determining the price.

*Participant 21, civil society*

### Medicine regulation

Patient access to novel medicines has become faster in recent years in South Africa, for the regulatory process has become more streamlined and has started to line up with best practices globally. Despite these improvements, participants expressed the hope to further expedite the process and reduce duplication of efforts through harmonisation with neighbouring countries or a reliance model with other international regulators.

Nonetheless, many participants indicated that key childhood cancer medicines are not registered in South Africa, which means they cannot be listed on the Essential Medicines List (EML), affecting pricing and reimbursement as well. In paediatric oncology, this is a multipronged problem: (1) there are significant problems with older but essential products being discontinued, (2) there is a lack of age-appropriate formulations and (3) newer products are never filed for registration due to the small market. To incentivize the registration of orphan drugs, there is a need for regulatory incentives such as expedited approvals, exemptions from importation requirements or tax reductions.

Section 21 of the Medicines and Related Substances Act 1965 (Act 101 of 1965) allows pre-registration access to medicines and thus provides an alternative access pathway. This access pathway was associated with many issues, including (1) a lack of transparency in their pricing, (2) exponential pricing for small patient groups due to post-importation testing and local packaging requirements, (3) limited compensation by insurers for these medicines and (4) considerable delays in acquiring products, which can be detrimental for childhood cancers. As Section 21 medicines are not subject to the SEP policy requirements, there are opportunities for discounting and price negotiations with manufacturers. One medicine supplier indicated that, hence, this access pathway is sometimes preferred for small patient populations.

If you've got a pediatric oncology patient or a patient that needs something tomorrow morning, if it's unlicensed, that's not happening. That process can be anywhere from two to four weeks, to get a product from the US or from Europe, into South Africa.

*Participant 19, policy-maker/regulator*

### Financing and pricing

With regard to budget allocation for health services, including medicine procurement, participants expressed concerns about allocated budgets based on what was historically assigned, as well as concentration of funds in one province (Western Cape). Healthcare providers also expressed frustration at the lack of transparency in the spending of the National Tertiary Services Grant (NTSG) – a grant that can be used by public sector hospitals to procure medicines outside of the EML – desiring clarity on the available budgets and where it is spent, if not on paediatric oncology. Furthermore, participants indicated a need for renewal of a temporary grant system to assist families with increased expenses due to cancer treatment. Participants had mixed feelings about medicine donations, being grateful for the support yet cautious about continuity of services when donations are discontinued.

High cancer medicine prices were broadly identified as a barrier. Some participants expressed incomprehension at the high prices since marketing of medicines on the South African market generally occurs much later than in Europe or the United States. The need for alternative reimbursement/payment models in the pricing of novel and orphan medicines was thus repeatedly voiced by stakeholders across the value chain. However, the lack of a Health Technology Assessment (HTA) and a responsible agency in South Africa was identified as an obstacle in achieving this. In the private sector, participants indicated a need for clarity around the definition of value and the role of HTA in reimbursement decisions, with consistency across medical schemes.

We know that we’re actually not the first country that’s prioritized for the introduction of a new molecule. […] But we are subjected to the same requirements by companies who say: ‘I need to recoup the money that I put into making this drug’. […] Firstly, but secondly, the purchasing power parity of the rand, what a rand buys to what a pound buys in the UK, those are two different things. But we find ourselves paying exactly the same prices, especially when it comes to oncology drugs. And we feel that is very much unfair.

*Participant 5, policy-maker/regulator*

### Selection

The Standard Treatment Guidelines (STGs) and associated National Essential Medicines List (NEML) are key in guiding the rational use of resources and medicines in the South African public sector. Childhood cancer medicines are used exclusively in tertiary or quaternary settings and therefore included in the tertiary/quaternary NEML. This list is – unlike the primary and secondary NEML – not accompanied by STGs. This was viewed as logical by health care providers, given that these medicines are solely prescribed by experts, whereas policy-makers voiced worries about the lack of accountability of services. Additionally, policy-makers and regulators indicated that the current tertiary/quaternary NEML is still adult-dominated, due to the limited attention that has been paid to (less prevalent) paediatric indications since the inception of the list. A thorough review of the completeness of the NEML was thus advised. Paediatric oncologists should be actively involved in this, whose engagement in and advocacy on this matter was previously felt to be missing by policy-makers and regulators. On the other hand, healthcare professionals expressed having difficulties in getting childhood cancer medicines on the NEML, as evidence for children is often more anecdotal and based on expert opinion. Current NEML evidence requirements do not cater for this and should be reviewed. However, even when medicines do make it on the NEML, expensive medicines in particular risk being designated as an unfunded mandate by individual provinces that lack the funds to cover their procurement. This threatens the sustainability of the NEML system. Finally, one participant advocated for oncology medicines to be classified as *vital* instead of *essential* because of their progressive nature, which could trigger faster responses from the procurement team in case of stock-outs.

So at the moment, a lot of policy is driven from a very strong evidence-based medicine perspective. […] Problem when you’re sitting around the table and trying to make decisions about pediatric oncology, is that that evidence base is largely derived from adults medicine, and quite often very weak compared to on the ground clinical outcomes.

*Participant 14, medicine supplier*

At the local level, concerns were raised about the decision-making of Pharmaceutics and Therapeutics Committees (PTCs). These committees govern medicine use at the provincial, district, sub-district and health facility levels and must approve the use of medicines outside of the NEML. Voiced concerns included (1) their lack of transparency and consistency in decision-making and spending, (2) the lack of expertise of committee members in paediatric oncology, which was felt to be necessary to understand why patients require medicines outside of the NEML, (3) duplication of efforts across provinces and hospitals, (4) high evidence requirements, which preclude approval and 5) the time and effort clinicians need to invest in preparing a motivation for a given medicine.

The representatives on the PTC is not very often oncologists. So if you’re not an oncologist, you’re not going to know how important the drug is that we are motivating for.

*Participant 24, healthcare professional*

### Reimbursement

Over 70 medical aid schemes are available in South Africa to assist with managing medical expenses within the private sector. However, civil society stakeholders indicated that many families with medical insurance do not have cancer benefits, having purchased only basic insurance plans (at so called prescribed minimum benefit (PMB) level) or not having purchased specific cancer coverage options. Treatment components that are almost always excluded from coverage include blood works, prosthetic limbs and palliative care. High co-payments may be imposed on families without cancer coverage, or once insurance benefits are exceeded. These may force members to leave their insurance plan and seek treatment in public sector facilities. Other families are forced to abandon their insurance because income was lost due to one parent needing to accompany the child during treatment.

In the private sector, it’s dependent on the medical scheme. And if they don’t have cancer benefits, then you’re stuck. And I mean, that’s the reality because the majority of people do not buy medical insurance with the perspective that my child is going to be diagnosed with cancer, so most people have hospital plans. So they have a basic and a hospital plan. And therefore they don’t have any of the plans that would cover cancer treatments, and cancer treatments in the private sector is horrifically expensive.

*Participant 21, civil society*

However, insurance schemes may opt to reimburse medicines and services not part of an individuals benefit package. These are called ex-gratia (or ad-hoc) payments. Participants had several concerns about the decision-making process for these payments, which include (1) the lack of clinical evidence that complicates decision-making, (2) that the extent to which clinicians and parents advocate for a medicine affects the outcome of the decision and (3) the lack of transparency and consistency in decision-making of insurance schemes. Medical scheme representatives indicated that they are sympathetic towards children and often offer (partial) coverage.

Several deficiencies in the regulation of insurance schemes, provided by the Council for Medical Schemes, were also revealed by participants. These included controversies over what must be covered by insurers, due to (1) unclarity when treatment is for cancer eradication versus palliative care and (2) private sector facilities pointing to standards of care in public sector facilities where treatment options may be more limited. In addition to this, reform was requested on (1) how PMBs are defined, potentially based on which services are essential versus which diagnoses are covered and (2) guidance of the Council for Medical Schemes to funders which medicines to reimburse, to increase consistency in the process and decisions towards reimbursement.

So the big issue is like, where is the treatment for cancer eradication versus palliative care, etc? Those are not necessarily fully defined exactly what should be covered, not covered. But because most medical schemes create financial limit, inevitably, it blurs the line.

*Participant 8, medical aid scheme representative*

### Supply and procurement

For the public sector, those medicines that are on the NEML are procured through national tenders. Participants indicated that although very low medicine prices are achieved through this system, not all products are successfully tendered due to a lack of bids. Medicine suppliers suggested that the State’s low price expectations can make bidding unprofitable. It was suggested that the possibilities for pooled procurement should be investigated to increase suppliers’ interest in the South African market, and by extension that of other countries in the region. Other reasons for unsuccessful tenders include low volumes needed by the state and too comprehensive requirements keeping suppliers from bidding. Instead, suppliers were criticised for pursuing more advantageous pricing through buy-outs. Other concerns about the tender process include the lack of accountability when suppliers are unable to supply what has been agreed upon, as well as misalignment between tender cycles – which run for two or three years – and the entry of new (generic) products on the market.

First of all, they don’t tender for it. If they do tender, then they actually at times give you delays in acquiring. […] I would say 80% of them are good. But the problem with chemotherapies is it comes in a package. And you can’t suddenly say I’ll use half of your package and the other half [not].

*Participant 3, policy-maker/regulator*

Medicines not successfully procured through the tender process and products that are not listed on the NEML must be acquired through a buy-out process. This process is organised provincially or locally. Buy-outs – needed regularly in paediatric oncology – are associated with several drawbacks according to participants. These include that (1) procurement can be delayed given that buy-outs take time, with insufficient stocks to bridge the interim, (2) provinces have limited budget to spend on medicine procurement, further complicating this process and (3) supply cannot be guaranteed for products not on contract. Given that childhood oncology treatment is often concentrated in major quaternary centres, the buy-out process is easier to navigate because these centres have bigger budgets and well-established contacts with suppliers.

On a local level, some healthcare providers indicated having reliable and timely procurement processes in place in their respective hospitals, whereas others expressed frustration at the lack of compassion and urgency from other members of the procurement chain when confronted with stock-outs. Participants speculated that not all members are aware that medicines on the NEML should always be available. Additionally, communication with the provincial medicine depot can be arduous, causing delays in procurement. Some healthcare providers also suggested that distribution systems are inadequate, with products not reaching treatment facilities or no ability to maintain cold chains.

Nowadays you just send it with the ambulance driver and you can’t even maintain like cold chains and things. There are no pharmacy courier services available anymore. So there’s no way of getting therapies in any reasonable time actually from one hospital pharmacy to another.

*Participant 26, healthcare professional*

As a result of these barriers, some healthcare providers reported recurrent stock-outs in their facilities, while others indicating having almost no shortages. If stock-outs occurred, it resulted in disrupted treatments or medicines omitted from treatment regimens. Although shortages can sometimes be solved by buying items from private sector facilities: (1) there is usually a delay of 4–5 days disrupting treatment and (2) private sector stocks are insufficient to supply the entire public sector. Besides shortages of chemotherapeutics, the availability of anti-emetics and palliative care medicines at pharmacies for use at home is often problematic.

### Healthcare delivery

Late detection of childhood cancers was stressed as a major contributor to poor outcomes in the public sector by all stakeholder groups. Several factors contribute to late diagnoses, including delayed health-seeking behaviour, poor recognition of symptoms by primary healthcare (PHC) workers and the general public, consequent misdiagnoses, and a lack of diagnostic tests performed at the PHC level. To achieve earlier detection, participants proposed that the general level of training at the PHC level requires improvement, including training on recognising cancer symptoms and doing bloodwork. Participants also suggested that overburdening of the clinics contributes to low motivation of personnel and missed diagnoses.

Further delays in diagnosis and treatment often occur due to the limited number of paediatric facilities and specialists in the country. Additionally, participants indicated interprovincial referrals – necessary because not all provinces have paediatric oncology units – can be uncertain in both the public and private sector. In the private sector, late detection of cancers may be due to parents passing over PHC level, and immediately seeking healthcare from specialists instead. Waiting times at hospitals may thus contribute to delayed diagnoses.

Participants highlighted a lack of healthcare professionals at all levels of care and from all disciplines, including paediatric oncologists, haematologists, specialised nurses and pharmacists, palliative care specialists and PHC workers. The shortages lead to overburdening, staff leaving, long waiting times, and untrained staff performing duties for which they received no training. The insufficient number of healthcare professionals is compounded by a lack of (formal) training opportunities for oncology pharmacists, paediatric oncology nurses and palliative care specialists, according to healthcare professionals themselves. Participants called for formal training platforms, formal accreditation for paediatric oncologists and haematologists, and continued monitoring and development of skills on the work floor.

First of all, pediatrics have got no nurse trained [in] pediatric oncology […]. And there’s no training platforms for them either.

*Participant 3, policy-maker/regulator*

As a pharmacist, I don’t like the fact that I’m teaching myself everything, there isn’t support in terms of equipping the people who are in the field. It’s tragic that I did not learn about pediatric oncology yet I’m expected to practice in it.

*Participant 25, healthcare professional*

Besides chemotherapy, other resources and disciplines are involved in diagnosis, treatment and follow-up care. Multiple barriers were identified in these. Firstly, healthcare professionals indicated that there is an increasing need for diagnostic and radiologic resources such as PET (positron emission tomography) and/or CT (computed tomography) scanners. Secondly, participants considered the mental health of children with cancer not to be a priority in the public sector, again compounded by overburdened staff. There are almost no government-provided social workers. Thirdly, a participant pointed out that there is historically little attention for palliative care, with (1) palliative care currently not being a recognised specialty, (2) little to no dedicated paediatric palliative care services available in hospitals and (3) insufficient coverage within medical aid plans. In fact, limited knowledge of healthcare professionals on palliative care even contributes to discontinuation of palliative care and insufficient stocks of morphine in pharmacies. Despite this, recognition for palliative care in oncology seems to be increasing.

On an organisational level, healthcare professionals pointed out that cancer services are centred around adults, with little recognition of the needs of children. Examples mentioned include the lack of oncology services over the weekend (although paediatric regimens can run 5–7 days), or children aged 12 and older being treated in adult wards.

### Dispensing

Several participants expressed concerns about the quality of pharmaceutical oncology care. In particular, the preparing and mixing of therapies performed without equipment or training is concerning. This is compounded by deficient Good Pharmaceutical Practice (GPP) guidelines. In addition to this, chemotherapeutics are regularly administered by individuals without appropriate training due to a lack of paediatric oncology nurses. Pharmaceutical care may also be compromised due to a lack of clinical responsibility of pharmacists, despite their involvement being considered crucial in paediatric oncology by healthcare professionals.

If you read the GPP *[Good Pharmaceutical Practice]* documents, you will see that it is non-committal. It allows a wide scope of practice. So what is happening at the moment in South Africa is the most of the mixing of chemotherapy is happening in doctor-driven practices in facilities that are not registered with the Pharmacy Council.

*Participant 14, medicine supplier*

### Use

A range of stakeholders highlighted the social barriers around childhood cancer treatment. Families’ inability to travel was considered an important one, given that distances to specialised treatment facilities can be far and travel costly. Establishing paediatric cancer care facilities closer to home was recommended by all stakeholder groups. Another social barrier identified was the generally delayed health-seeking behaviour, because symptomatology may be accepted as part of life. This was indicated as a complicating factor to late diagnoses. Other parents may be late to bring their child to a health facility because they seek help from traditional healers first. Finally, cancer treatment may considerably disrupt the life of the child and its family on multiple levels, due to missing school, fragmented families for an extended period of time, and lost earnings by the caregiver accompanying the child.

Potential defaulting on treatment was also identified as a user barrier. Healthcare professionals and civil society stakeholders attested that defaulting happens occasionally, due to (1) resistance to surgeries, particularly inoculations and amputations, (2) caregivers’ believe that the child has been cured when it shows signs of improvement and (3) financial constraints. Some abandon chemotherapy treatment temporarily to consult a traditional healer, significantly contributing to poorer outcomes. Multisectoral support by non-profit-provided social workers and invested physicians – as well as financial aid – can help to prevent defaulting.

Patients do not even have money for food. So they would rather concentrate on what is essential than getting the child to the hospital and spending money on transport to the hospital.

*Participant 24, healthcare professional*

### Monitoring and surveillance

Participants indicated little resources being allocated to monitoring and surveillance, also demonstrated by an inaccurate cancer registry. Electronic instead of paper-based healthcare records were proposed as a means to facilitate surveillance and identify missed diagnoses. More accurate prevalence rates could contribute towards easier price negotiations and increased access.

So in the Western Cape, they’re building an electronic health record. You can see somebody was seen at a clinic. Somebody could look at this from the outside and go, hang on that doesn’t look right. In a rural area, that paper record is inaccessible. And we don’t know who’s being missed, nobody is checking.

*Participant 2, policy-maker/regulator*

### Emerging cross-cutting themes

Several themes emerged as intersecting barriers across multiple components of the pharmaceutical value chain. These included:

#### Advocacy

A range of stakeholders – including policy-makers – indicated the need for advocacy by clinicians and organisationsNGOs on numerous issues. These included (1) the manufacturing or marketing of (discontinued) medicines, (2) inclusion of essential childhood oncology medicines in the NEML, (3) lack of clinical standards for paediatric oncology, (4) coverage of childhood oncology medicines within the PMBs and (5) policy development for childhood cancers and rare diseases in general. However, despite its emphasised importance, there were opposing views on whether adult and child oncology advocacy should be unified or separated. Additionally, civil society stakeholders considered their advocacy efforts effective, whereas policy-makers and regulators expressed that they had not picked up on any advocacy for paediatric oncology.

#### Awareness

All stakeholder groups emphasised the need for increased awareness of childhood cancers, among PHC workers, traditional healers, early childhood development systems (schools and baby clinics) and the general public. In like manner, there was felt to be insufficient awareness on (1) referral pathways among patients, (2) Section 21 access among prescribers and patients, (3) medical aid rules among members and (4) bone marrow transplants among possible donors.

#### Equity

All stakeholder groups pointed out persistent inequities in (access to) childhood cancer services, between (1) urban and rural areas, (2) between the public and private sector, (3) between facilities – within both the private and public sector, (4) between medical aid schemes and (5) between provinces.

#### Non-governmental organisations

Healthcare providers and civil society stakeholders highlighted the importance of NGOs in the paediatric oncology field, by providing numerous essential services and support, including (1) awareness programmes among PHC workers, traditional healers and general public, (2) travel aid, accommodation, food packages and toiletries for public sector parents, (3) wheelchairs and pressure mattresses for at home, (4) financial assistance to pay medical aid member fees and co-payments, (5) monetary support for prosthetics and (6) psychosocial support services. However, civil society stakeholders experienced financial challenges as they are not supported through government funding, despite providing several essential services.

## Discussion

Health systems research is vital to inform policy development and advocacy efforts for childhood cancer in LMICs, enabling the identification of barriers to childhood cancer care and facilitating targeted health system improvements to address them effectively (Denburg et al., 2014). We performed such a comprehensive health systems analysis of determinants of access to medicines used in childhood cancer in South Africa, providing context-specific data on how various national pharmaceutical processes contribute to access. Key issues in accessing medicines – noted by multiple stakeholders or indicated as major barrier by stakeholders – include (1) a lack of political priority given to childhood cancer (medicines), (2) no registration of novel drugs as well as discontinuation of traditional chemotherapeutics from the market, (3) incomplete insurance coverage for childhood cancers and (4) (intermittent) stock-outs of essential medicines. However, broader health system determinants relevant to childhood cancer care were also identified, including low awareness on childhood cancers among PHC staff and the general public, and patients’ inability to access care facilities. The need for flexibilities in the SEP policy, regulatory incentives for orphan medicines, transparency in decision-making processes and healthcare spending, and improved training of PHC staff, nurses and pharmacists in paediatric oncology emerged as priority interventions to improve childhood cancer medicine access and equity in South Africa.

In an LMIC still battling high rates of HIV and TB (Institute for Health Metrics and Evaluation, n.d.), the lack of consideration given to childhood cancers – also compared to more common adult cancers – is not unexpected, yet remains highly problematic due to the aggressive nature and unique treatment requirements of paediatric cancers. Limited policy commitment for paediatric oncology was also observed in other countries on the African continent (Boateng et al., 2020; Petricca et al., 2023), and LMICs elsewhere (Boateng et al., 2021). Interviews with stakeholders revealed that limited attention to childhood cancers is encountered in all stages of the South African pharmaceutical value chain, from the policy arena to the organisation of healthcare services and among cancer advocacy organisations. The rarity of these diseases further complicates the lack of priority given to them. In a country without an official definition of what a rare disease entails, a new way of dealing with these type of diseases is urgently required: from targeted policy development, to creating regulatory incentives for small patient populations, tailored pricing solutions and HTA, adapting evidence requirements by decision-making bodies (the NEML committee, PTCs, and for ad-hoc payment decisions) as well as reflecting childhood cancer treatment modalities – including palliative care – in PMBs.

However, in a country with limited legislative capacity, the present focus on achieving NHI leaves little room for addressing the deficiencies in existing healthcare policies, despite the evident need and desire for it (Naidoo et al., 2023). Still, other possible interventions that do not require major legislative changes exist and include the need for (improved) training platforms for PHC staff, nurses and pharmacists in paediatric oncology, more clarity on regulations in the private funding environment by the Council for Medical Schemes, increased transparency in decision-making processes, revising GPP guidelines and expansion of existing awareness efforts (van Heerden et al., 2023). Internally, provinces and hospitals should address known inefficiencies in procurement (Magadzire et al., 2017; Modisakeng et al., 2020) and educate their personnel where necessary.

Interviews with stakeholders from different provinces and hospitals again highlighted the differences between some of the centres of excellence in the provinces of Western Cape and Gauteng compared to the other treatment facilities in the country (van Heerden et al., 2021). While some participants reported having well-organised supply systems and no problems acquiring products outside of the NEML, others faced significant obstacles in getting access to products that are not on the NEML. This barrier may apply to more novel, biological therapeutics in particular, with the majority of traditional chemotherapeutics already listed on the NEML (Joosse et al., 2023). Nonetheless, the regional disparity in access highlights the importance of the NEML in providing access to medicines. The apparent lack of engagement from some of the specialised treatment centres in the NEML is thus particularly detrimental for some of the smaller units, which would benefit from the large quaternary hospitals taking the lead in improving these core structures and advocating for more essential childhood cancer medicines on the NEML. This could also reduce the number of medicines that need to be bought out (Modisakeng et al., 2020) increasing the affordability and sustainability of the system as a whole. Additionally, since the expansion of services – especially by establishing new paediatric cancer units in provinces where there are currently none – was reiterated as an important intervention to increase access, a more inclusive NEML would benefit novel treatment units as well.

An important strength of this study is the number of stakeholders interviewed (n = 29), together representative of a wide range of stakeholders. This complete analysis of the South African pharmaceutical value chain has identified known (van Heerden et al., 2021, 2023; Magadzire et al., 2017; Mattila et al., 2021; Meyer et al., 2021, 2022; Modisakeng et al., 2020; Stefan & Siemonsma, 2011) and unknown weaknesses in the system, providing a comprehensive overview of the barriers and facilitators to access. Although our aim was to obtain a better understanding of how various pharmaceutical processes contribute to the inaccessibility of childhood cancer medicines, the interviews brought forth broader health system barriers. Given the tight link between access to medicines and broader care delivery – particularly in cancer care – these factors have been incorporated in the overview. To which extent the identified barriers may impact adults was not specifically studied, but we infer that several factors likely affect the general population as well. Additionally, we did not study international drivers of access (such as international market forces, global shortages, and R&D and innovation) which undeniably affect access on a country level. Similarly, this study primarily revealed barriers to curative services rather than palliative care, which is associated with particular challenges (Makoni, 2021) and warrants further systematic study. Furthermore, despite the significant number of participants, our sample did not include participants from all nine South African provinces. With that, this study is not an exhaustive comparison of regional barriers but rather an analysis of the entire system, indicative of the systemic issues at play. Finally, despite efforts to limit participant and researcher biases (i.e. introducing the study, establishing rapport with participants, asking probing questions, using a standardised interview guide), these biases are inherent to this type of research and cannot be completely eliminated.

## Conclusion

This is the first comprehensive study of determinants of access to medicines used in childhood cancer in South Africa, adding to a growing evidence base on access to childhood cancer medicines in LMICs. The substantial number of – larger or smaller – barriers identified across the pharmaceutical value chain suggests that a step-wise approach is needed to address the issues. The context-specific evidence generated can enable appropriate policy development and advocacy efforts for improved access to childhood cancer medicines and reduced health inequities.

## Supplementary Material

Supplemental Material
