# Supplementary material for: Access to childhood cancer medicines in South Africa: a health systems analysis of barriers and enablers
Source: J Pharm Policy Pract. 2024 Jul 12;17(1):2372033. doi: 10.1080/20523211.2024.2372033 (PMC11249159; doi:10.1080/20523211.2024.2372033)
Supplement: Supplemental Material [file JPPP_A_2372033_SM7662.docx]

# Supplementary materials

## Appendix 1

**Interview guide**

*[Introduction]*

**Policy environment or governance.***[This theme captures the means by which childhood cancer services are organized, managed, and regulated. It includes topics such as the policy environment, priority setting and clinical standards.]*

What can you tell me about the policy environment for childhood cancers?

Do you know of any policies being developed to improve access?

Is childhood cancer a priority in your view? What do you think about that?

What is your view on clinical standards for childhood cancers?

What do you think are barriers or facilitators to access here in your experience?

How do you think the policy environment is different for childhood cancer medicines versus medicines for children and adults in general?

**Financing**

*[Financing encompasses the generation, pooling, and allocation of collective funds to cover childhood cancer medicines, as well as the methods of payment for individuals and organizations involved in childhood cancer care.]*

Can you tell me about how the funds for childhood cancer medicines are generated?

Can you tell me about how the funds for medicines are distributed and how this impacts access to these medicines?

Probes:

- Innovative financial instruments
- coverage for childhood cancer; special access programs
- compensation for childhood cancer
- payment methods

What do you think are barriers or facilitators to access here in your experience?

How do you think financing is different for childhood cancer medicines versus medicines for children and adults in general?

**Social aspects of care***[This theme includes population characteristics and health behavior.]*

What is your view on social determinants and access and adherence to cancer medicines?
Probes: income level, distance to health facility, patient characteristics.

What do you think are barriers or facilitators to access here in your experience?

How do you think social aspects are different for childhood cancer medicines versus medicines for children and adults in general?

**Medicine delivery**

*[This theme covers the structures, resources, and medicines required for the direct provision of care.]*

Can you tell me about the procurement, storage and distribution of childhood oncology medicines and how this impacts access to these medicines?

Can you tell me about the availability, prescription and use of these medicines and how this impacts access to these medicines?

What do you think are barriers or facilitators to access here in your experience?

How do you think medicine delivery is different for childhood cancer medicines versus medicines for children and adults in general?

*[Closure]*

## Appendix 2

**Participant characteristics**

**Table S1** Baseline characteristics of participants

| **Group** | **Participants (n)** | **M/F (n)** | **Age  (median; range)** | **Years of experience (median; range)** |
| --- | --- | --- | --- | --- |
| **Policy makers and regulators** | 7 | 3/4 | 57 (38-62) | 25 (7-41) |
| **Medical schemes** | 5 | 2/3 | 50 (36-61) | 25 (11-28) |
| **Medicine suppliers** | 7 | 5/2 | 42 (33-58) | 11 (5-30) |
| **Civil society** | 4 | 1/3 | 48 (43-62) | 16 (6-25) |
| **Healthcare providers** | 6 | 2/4 | 48 (34-66) | 17 (2-37) |
| **All** | **29** | **13/16** | **49 (33-66)** | **19 (2-41)** |

M = male, F = female

## Appendix 3

**Selected stakeholder quotes for identified barriers and facilitators.**

**Table S1** Selected stakeholder quotes

| **POLICY AND LEGISLATION** | |
| --- | --- |
| **Policy and legislative environment** | |
| **Lack of political priority** | |
| Policy maker/ regulator | “It's [pediatric oncology] such a small area that often I do think it gets neglected as being thought of as something on its own. We always try to consider pediatric oncology when we're looking at the general oncology space, but it doesn't always fit nicely into that kind of basket.” |
| **Policy to implementation gap** | |
| Medicine supplier | “My concern with South Africa is that we are very good at writing policy documents, we are very good at saying what we want. However, when it comes to implementation, it's quite slow.” |
| Civil society | “There are a lot of things that can be done in terms of law, policy, setting up institutions or units within government. But all of that is hamstrung by lack of state capacity.” |
| **Lack of multi-stakeholder engagement** | |
| Medicine supplier | “There's a huge need for government and the private sector – and here the private sector we talk about funders, physicians and the pharma industry – to have actual dialogues. And I think this is something which is missing. And I don't like to say it, but it generally is because government is extremely difficult to nail down, to get to a meeting, or to understand who the right level of stakeholder is.” |
| Policy maker/ regulator | “I think in engaging government […], at least from my experience what we find is that from the stakeholders like your pharma industry stakeholders, they engage government from a combative point of view. Which does not help anybody, because everybody then ends up being defensive.” |
| **Current pricing policies** | |
| **Problems with Single Exit Price (SEP) policy** | |
| Civil society | “In the single exit pricing system, there is, as far as I'm concerned, no transparency, even though government sees it as a transparent system. What is transparent is the price. Yes, we advertise the price. But it's still the pharmaceutical company that is actually deciding and determining the price.” |
| Medicine supplier | “So the two biggest barriers for us is reference pricing [by other countries], international benchmark pricing, and the SEP. Because our SEP is visible, and it's published on a public website, and everybody [other countries] can access that.” |
| **Medicine patent laws** | |
| **No examination of patents** | |
| Civil society | “So there are issues in patent law in South Africa that... […] South Africa grants many more patents than comparable countries like India and Argentina. And there's been comparisons done, that show this, we grant an inordinate amount of patents, often poor quality, secondary patents. And that's due to various shortcomings in our law. […] But we don't examine patents, we grant them and then they get appealed. […] So that's one thing once a patent is granted, it's very hard in our legal framework to overturn the patent.” |

| **REGULATION** | |
| --- | --- |
| **Regulatory process** | |
| **Regulatory process has improved** | |
| Healthcare professional | “The regulatory process here used to be quite long and laborious, but it's become fairly streamlined, so the average time to registration of a generic item is about 250 days and for a new chemical entity about 500 days. So, it's fairly expedited compared to the past.” |
| **More regulatory efficiency needed** | |
| Medicine supplier | “The hope – obviously – is that with our regulator we can also move to sort of a reliance model, where the South African regulator can start looking at how the first launch country regulators – like the FDA or the European Union – are dealing with these things, and hopefully piggyback off of a lot of that. |
| Civil society | ”And I guess one of the solutions that people keep talking about is kind of regulatory harmonization? Which I think is critically important.” |
| **Registered products** | |
| **Products discontinued or not registered** | |
| Healthcare professional | “I think biggest barriers for us are, obviously, the fact that some of your medication are just not registered for whatever reason that may be.” |
| Policy maker/ regulator | “I mean we constantly get companies who are discontinuing medications because they're just not a… it's just not economical for them.” |
| Healthcare professional | “There's no child friendly formulations [available], right? That is like, we are always trying to find our way around. Even etoposide capsules, it's a 50 milligram capsule, children sometimes need less than that. Even getting the capsule is like finding a piece of gold.” |
| **Alternate access pathways** | |
| **Issues in pricing and procurement of section 21 medicines** | |
| Medicine supplier | “If you've got a pediatric oncology patient or a patient that needs something tomorrow morning, if it's unlicensed, that's not happening. That process can be anywhere from two to four weeks, to get a product from the US or from Europe, into South Africa.” |
| Medical scheme | “When you do that [section 21 access], then you have to go and procure outside of the country, and you have to find who's going to deliver the best price. […] Was that the cheapest price or not?” |
| **Loophole in pricing for section 21 medicines** | |
| Medicine supplier | “Section 21, which allows you pre-registration access, is probably the best way to deal with this [high molecule costs] long term. Because unlike once you get it registered when we have to have a single exit price which becomes visible to the rest of the world, your section 21 is not visible. So for small patient numbers, you can actually come up with a pricing solution which is more relevant to maintaining access for those patients.” |
| **Regulatory incentives** | |
| **Need for incentives or exemptions** | |
| Policy maker/ regulator | “I think we probably have to think out the box in terms of orphan diseases, that kind of priority, how do you prioritize rare pediatric cancers? You know, is there a tax break, is there a VAT, do you have a regulatory environment that is quick through the process to do that, how do we make it viable for that? I think that's not been well discussed.” |
| Medicine supplier | “To have products available in local packs is a barrier, because it's quite costly and increases the cost price of medicine and then eventually the SEP. So if we can have exemption from those requirements, I think there will be a definite increase in access.” |

| **FINANCING AND PRICING** | |
| --- | --- |
| **Budget allocation** | |
| **Concerns about allocated budgets** | |
| Policy maker/ regulator | “And our budget, it's not at the moment much… it’s more historically assessed. Oh you spend so much, so I give you 10% more. They don't look at what is needed and what the gap is, you see.” |
| **Funding of tertiary services** | |
| **No transparency in spending** | |
| Healthcare professional | “There's this national tertiary grant that we can access. But I don't know what's happening with this national tertiary grant. […] Can we access this NTS [National Tertiary Services]? Is this NTS budget available? Because we should be able to access this national tertiary grant, if the budgets elsewhere is being exhausted.” |
| **Care grants** | |
| **No temporary care grants** | |
| Healthcare professional | “In the past, we could apply for grants for our patients. So while they are on treatment, we apply for the grant, for them to at least have transport money to get to the hospital, we could actually motivate for the grant to be renewed every two years or every three years. And once a treatment is completed, we can say stop the grant. That system has completely fallen away.” |
| **Medicine donations** | |
| **Non-sustainable system** | |
| Policy maker/ regulator | “It's often a difficult avenue though, to kind of manage I think, and I mean I think – I know it's bad to say – but often we look at donations with a little bit of skepticism, because they are great for a period of time and then they come to an end and then the service that you were providing, now all of a sudden you need to either find funds to cover it or all patients then go without the service, so that's just one of the concerns in that space.” |
| **Medicine prices** | |
| **High prices of medicines** | |
| Policy maker/ regulator | “We know that we’re actually not the first country that's prioritized for the introduction of a new molecule. […] But we are subjected to the same requirements by companies who say: “I need to recoup the money that I put into making this drug”. […] Firstly, but secondly, the purchasing power parity of the rand, what a rand buys to what a pound buys in the UK, those are two different things. But we find ourselves paying exactly the same prices, especially when it comes to oncology drugs. And we feel that is very much unfair.” |
| **Innovative financial instruments** | |
| **Alternative reimbursement/payment models needed** | |
| Policy maker/ regulator | “If you want to look to attract, then you need to have innovative modeling in terms of how you would consider payment, what your reimbursement models are, we need innovative reimbursement models in less prioritized diseases, various diseases, we need to find how to do that. And so that's a leap. That's, that's a new language.” |
| **No formal HTA process and guidance** | |
| Medicine supplier | “If we had some sort of independent organization who could look at these and give guidance to both the government and the private sector, that makes sense. What I don't like at the moment is because the government don't really ask for it, we just use the HTA as almost a value add when you're trying to go into a tender process or buy-out process. In the private sector, every funder wants you to submit an HTA to them, and each of them charge you a fee for reviewing it. But yet, they don't have a outcome in terms of what that means for reimbursement.” |

| **SELECTION** | |
| --- | --- |
| **Essential Medicines List (EML)** | |
| **Review of EML needed** | |
| Policy maker/ regulator | “The kick starter has been adult medicine for whatever it was, adult oncology, adult cardiology, it was always going to be adult because that were the big users. And so it's only now this realization that pediatric psychiatry is important, or pediatric neurology and that... So I think there's a lot of catching up to do to inform decision-making on the EML. And so I think we're very behind on that.” |
| **High evidence requirements** | |
| Medicine supplier | “So at the moment, a lot of policy is driven from a very strong evidence-based medicine perspective. […] Problem when you’re sitting around the table and trying to make decisions about pediatric oncology, is that that evidence base is largely derived from adults medicine, and quite often very weak compared to on the ground clinical outcomes.” |
| **Need for classification of oncology medicines as vital** | |
| Policy maker/ regulator | “We've been fighting a long time asking for these drugs to become vital on the VEN [vital – essential – necessary] analysis for WHO. We think it's so important, it shouldn't be essential, it should be vital, like adrenaline or like any of those drugs. Because, one, the cancer is growing when you don't treat. Number two is, if you treat ineffectively, you don't get the responses you're supposed to get. And so these are challenging endpoints that are contributing significantly to the poor outcomes that we have within the developing world.” |
| **Unfunded mandates for new EML additions** | |
| Civil society | “And then all the province will just come back and say it's an unfunded mandate, meaning you've put it on the essential medicines list. So yes, it may be on the EML but sorry, either me or the province don't have the money to actually buy it.” |
| **Standard Treatment Guidelines (STGs)** | |
| **Lack of treatment guidelines for childhood cancers** | |
| Policy maker/ regulator | “It [lack of STGs for childhood cancers] makes it difficult to hold health systems to account to a standard. If something is on the essential medicines list and there's a clear standard treatment guideline, it's very easy to point to a lack of service and say that’s what is guaranteed, and you're not doing it. When you've just got a [tertiary essential medicines] list without details, it’s open to interpretation.” |
| Policy maker/ regulator | “The reason why we don't create STGs for tertiary drugs, is the fact that these drugs are being utilized by specialists. And because they're being utilized by those people, you need to know your work to get to that level. And so it doesn't make sense to us to create, and to include them into any document.” |
| **Pharmaceutics and Therapeutics Committee (PTC)** | |
| **Concerns about decision-making of PTCs** | |
| Policy maker/ regulator | “So each PTC meeting, it's confidential and there isn't clear reporting. What was paid for, what wasn't paid for, why it wasn't paid for and how much was even paid. So we aren't very good at transparency of expenditure in the public sector.” |
| Healthcare professional | “Because the provincial PTC, half the time they are not meeting often, especially during COVID they didn't have any meetings. Or the process stops there just because they would say ‘not enough evidence’ or ‘too expensive’. It's not vital.” |
| Healthcare professional | “The representatives on the PTC is not very often oncologists. So if you're not an oncologist, you're not going to know how important the drug is that we are motivating for.” |
| Healthcare professional | “There's so much duplication of what we [all provincial PTCs] are doing.” |

| **REIMBURSEMENT** | |
| --- | --- |
| **Coverage** | |
| **Incomplete coverage** | |
| Civil society | “In the private sector, it's dependent on the medical scheme. And if they don't have cancer benefits, then you're stuck. And I mean, that's the reality because the majority of people do not buy medical insurance with the perspective that my child is going to be diagnosed with cancer, so most people have hospital plans. So they have a basic and a hospital plan. And therefore they don't have any of the plans that would cover cancer treatments, and cancer treatments in the private sector is horrifically expensive.” |
| **Members forced to leave insurance plan** | |
| Civil society | “Most cases, I find it's because the one parent has to leave their jobs. So the income has either been cut in half or has like busy financial needs.” |
| **Grey areas in what must be covered** | |
| Medical scheme | “So the big issue is like, where is the treatment for cancer eradication versus palliative care, etc. Those are not necessarily fully defined exactly what should be covered, not covered. But because most medical schemes create financial limit, inevitably, it blurs the line.” |
| Medicine supplier | “Now, the prescribed minimum benefit, fine as a concept, that really points to the standard of care which is available in the public sector. And what we've all seen, I mean, the standard of care in the public sector, unfortunately, is going nowhere. […] So that has a direct and maybe a convenient consequence for the private funders in saying when we start bringing in innovation around, for example, rare diseases or new innovations in oncology, they don't have to pay for it.” |
| **Regulation of insurance schemes** | |
| **More clarity in regulation needed** | |
| Medical scheme | “The way that the regulations are structured, medical scheme benefits are determined based on the size of the wallet that they come with. And I think that's unfortunate. […] So yes, you need a prescribed minimum benefits that's probably more clearly defined, and we’re talking about it must not be defined on the basis of diagnosis, but on the basis of essential services.” |
| Medicine supplier | “And our frustration, I guess, is we have to negotiate with around 80 independent funders, all of them with different scheme designs, trying to figure out how you can actually bring a molecule to patients. And I think what would be really useful is the Council for medical schemes, who regulates the private funders, if they could make bring clarity around that process towards reimbursement.” |
| **Ex gratia payments by insurance schemes** | |
| **Concerns about decision-making** | |
| Medical scheme | “And a lot of times it's a lack of clinical evidence, a lack of trials with regards to the drug that they're asking for. You know, especially in the younger lot of children, like under a year or two years of age, we find that even where we do want to help and pay, there's nothing to base a particular decision on.” |
| Medical scheme | “If you look at cancer, it is an emotive condition. So, the extent to which medical aid are flexible in doing that [ad-hoc payments], it depends on the amount of pressure that is being put on. In this case, it would be the pressure by the member as well as the treating physician” |
| Policy maker/ regulator | “They [PTC meetings] are behind closed doors. Then each one is an individual case, they don't set precedent and they are not open about the reasons for their decisions. It's non transparent, but it could also be variable.” |
| **Sympathetic for children** | |
| Medical scheme | “We tend to be sympathetic where there is absolutely no other treatment alternative.” |
| **Price negotiation power of insurance schemes** | |
| **Successful price negotiations with medicine suppliers** | |
| Medical scheme | “If you [pharmaceutical company] want access, you need to bring it down to this [price] level. If you get more patients than this number, you need to gradually reduce the price. So that strategy sometimes works, especially on the PMB medicines.” |

| **SUPPLY AND PROCUREMENT** | |
| --- | --- |
| **National tenders** | |
| **Process issues** | |
| Policy maker/ regulator | “First of all, they don't tender for it. If they do tender, then they actually at times give you delays in acquiring. […] I would say 80% of them are good. But the problem with chemotherapies is it comes in a package. And you can't suddenly say I'll use half of your package and the other half…” |
| Medicine supplier | “Maybe they didn't meet certain requirements, because there's many requirements for the tender in terms of credit ratings, or good standing with the Department of Health, or maybe they don't have a BEE [Black Economic Empowerment] certificate. […] There's many other requirements within a tender that could render your submission invalid.” |
| Healthcare professional | “Companies who have the state tender, have to supply the medicine based on the contract they have. But when they can't, they're meant to provide within 14 days, but a lot of them don't. And no one ever holds them accountable.” |
| **Very low prices achieved** | |
| Medicine supplier | “With the tenders in the public sector, I think that's pricing that nobody can compete with. I think the companies go in so low on those pricing, that actually greatly affects how many people they [public sector] can actually assist with.” |
| **Procurement** | |
| **Well-organized supply system** | |
| Healthcare professional | “We don't have those barriers as a major problem in terms of procurement, things are in place where all of that gets done by the pharmaceutical side of the hospital.” |
| **Perceived lack of understanding from procurement team** | |
| Healthcare professional | “The buyout has to be done in what they call PPSD [Provincial Pharmaceutical Supply Depot]. […] So and very often that is where the problems occur because very often, we [physicians] don't have access to them [PPSD]. They don't communicate with us. They don't really know what is happening on the ground here. They don't know what the physicians face.” |
| **Alternative procurement strategies needed** | |
| Civil society | “Because they say the [South] African market is too small. That's why I'm coming back to pooled procurement across regions. […] If you are able to actually pool for sub-Saharan Africa, there’s a bigger market, there's a bigger possibility.“ |
| **Buy-outs** | |
| **Concerns about buy-out process** | |
| Policy maker/ regulator | “As soon as there's no tenders then you have to buy these drugs on an open market system. And nobody can guarantee supply because it's not contracted.” |
| Policy maker/ regulator | “These are the things, the barriers that do then impact the care of the child because if you're waiting two to three months or something, you're not sure how the child is going to be.” |
| **Acquiring through buy-outs easier for quaternary centers** | |
| Healthcare professional | “Most of the hospitals where we treat these patients are quaternary. And I think all of them are affiliated to a university. So it has its advantages in the sense that we've got the budget for acquiring and buying most of the medications that we need without any major problems.” |
| **Availability of medicines** | |
| **Unavailability and stock-outs** | |
| Policy maker/ regulator | “I think the other thing is that you put all your money in one basket. If that company goes, you've got nowhere to order from. Or if there are companies that do make it, but they don't make the quantities we want, because we [State sector] service about 75-80% of the population. So you will get these company, the other companies, they make quantities for the other 20%. So when you run out, they cannot supply, they cannot supply you.” |
| **Distribution** | |
| **Poor distribution systems** | |
| Healthcare professional | “Nowadays you just send it with the ambulance driver and you can't even maintain like cold chains and things. There are no pharmacy courier services available anymore. So there's no way of getting therapies in any reasonable time actually from one hospital pharmacy to another.” |

| **HEALTHCARE DELIVERY** | |
| --- | --- |
| **Diagnosis** | |
| **Lack of diagnostic capabilities** | |
| Policy maker/ regulator | “A lot of our children are coming at a late stage of disease, rather than at an early stage. And that's compounded by many factors. One is poor recognition of the clinical presentation [at primary healthcare center level], two is lack of standards to do the test.” |
| **Referrals** | |
| **Inadequate referral systems** | |
| Policy maker/ regulator | “Another barrier is just the uncertainty of how our tertiary services are organized. So at the moment some province is not going to have [public sector] tertiary hospitals at all. How do they get their patients to another province is a little uncertain. There’s meant to be interprovincial transfers and accounting for that, it's not working.” |
| Policy maker/ regulator | “And the referrals aren't that easy because of the limited amount of clinics and specialists available, so the delays will be there. And the costs I think incurred by parents for this are quite large.” |
| **Primary healthcare (PHC)** | |
| **PHC level training in public sector inadequate** | |
| Medicine supplier | “There’s lack of education at the primary clinics about cancers that needs to be… so the primary health care workers need to be upskilled on identification of cancers, on doing bloodwork.” |
| **Lack of use of primary care in private sector** | |
| Medical scheme | “Unfortunately, in the private funding environment, the emphasis has been put on referred care hospice, it's more referred and hospi[tal] centric. And the emphasis is not significantly on the primary care. So when you're talking about childhood [cancer], where there's limited screening, that first point of care becomes absolutely critical […], so your providers are well trained to be able to pick up early signs of potential problems.” |
| **Availability of staff** | |
| **Lack of healthcare professionals** | |
| Policy maker/ regulator | “There's only so many specialists within the country, so patients have to travel or are limited to go into these particular clinics where they have long waiting times, dates that are really far in the future.” |
| Healthcare professional | “Our young oncologists, or even very experienced staff, is leaving the country and there's a brain drain.” |
| **Training of staff** | |
| **Lack of training and formal accreditation** | |
| Policy maker/ regulator | “First of all, pediatrics have got no nurse trained [in] pediatric oncology […]. And there's no training platforms for them either.” |
| Healthcare professional | “Because there's no formalized training, it's up to individuals [palliative care specialists] to upskill themselves.” |
| Medicine supplier | “And there is no formal program in South Africa for pharmacists to obtain oncology pharmaceutical care.” |
| Healthcare professional | “As a pharmacist, I don't like the fact that I'm teaching myself everything, there isn't support in terms of equipping the people who are in the field. It's tragic that I did not learn about pediatric oncology yet I'm expected to practice in it.” |
| **Other resources** | |
| **Lack of diagnostic and radiology resources** | |
| Policy maker/ regulator | “Suppose radiotherapy units in the public sector, it’s an increasing problem, and so oncology patients – adult and pediatric – are battling to get access either to diagnostics or to radiological intervention.” |
| Medicine supplier | “There's also barriers with diagnostic testing. So there's often, we don't have that the tests available or we don't have PET scans available in the public sector or they’re out of commission.” |
| **Psychosocial care** | |
| **Lack of psychosocial support** | |
| Civil society | “They are so overwhelmed, the staff, with the amount of children that they have in [public sector] hospital that I don't think the mental health is such a priority.” |
| **Palliative care** | |
| **Lack of priority** | |
| Healthcare professional | “And palliative care was just a massive gap that we just haven't paid enough attention to. And it's suddenly starting to get the recognition that it needs.” |
| **Limited knowledge** | |
| Healthcare professional | “So morphine is technically available at every hospital, [but] it's not ordered by a pharmacy, because it's not always prescribed by the doctors, because there's still such a gap in knowledge around morphine. You know, if a child presents and they're on morphine, one of the first things they do is stop the morphine because it must be the morphine that's causing whatever.” |
| **Complementary and traditional medicine** | |
| **Delays in care or defaulting** | |
| Healthcare professional | “There's a strong cultural belief still in tradition. They first seek the help of a traditional healer […], and it takes sometimes – often – very long for them ultimately to get to us and by the time they get to us, it's too late. The cancer is at a stage four.” |
| Healthcare professional | “But it can also mean that they default treatment to go and seek an alternative. Because a lot of the patients we work with don't actually believe that cancer is really cancer, they think that it's a manifestation of an ancestor being unhappy.” |
| **Organization of services** | |
| **Child-specific cancer services not catered for** | |
| Healthcare professional | “The second thing is: pediatric regimens run on average five to seven days. In a state facility we operate, we function Monday to Friday, there is no oncology service provided over the weekend. […] So there is no on-call service for oncology, because the adults don't get chemo over the weekend.” |

| **DISPENSING** | | | |
| --- | --- | --- | --- |
| **Preparing and administering** | | | |
| **Gaps in safe and controlled preparing and administration** | | | |
| Medicine supplier | “If you read the GPP [Good Pharmaceutical Practice] documents, you will see that it is non-committal. It allows a wide scope of practice. So what is happening at the moment in South Africa is the most of the mixing of chemotherapy is happening in doctor-driven practices in facilities that are not registered with the pharmacy Council.” | | |
| **Pharmacist as healthcare provider** | | | |
| **Lack of clinical responsibility of pharmacists** | | | |
| Healthcare professional | “Here a pharmacist has got no clinical responsibility for a patient, they are making decisions around availability and not mixing therapies and whatever. But in theory, they are never around the table [with other healthcare professionals], having that clinical input. And just responsibility. And again, in pediatric oncology, isn't that essential?” | | |
| **USE** | | | |
| **Access to care** | | | |
| **Inability to travel** | | | |
| Policy maker/ regulator | | | “Time, lost earnings. You know. Who's going to accompany the child. Can they afford that? Massive social issues around that.” |
| Healthcare professional | | | “Patients do not even have money for food. So they would rather concentrate on what is essential than getting the child to the hospital and spending money on transport to the hospital.” |
| Civil society | | | “It [childhood cancer care] is quite centralized as well, which was quite a big challenge for people and you know, you have to travel 100 kilometers or 300 or whatever kilometers to go to the place where you get your cancer treatment.” |
| **Symptomatology accepted as part of life** | | | |
| Policy maker/ regulator | | | “The population generally, coming from the legacy of a party and of the past, are not people that go to doctors on a drop of a hat. They often go much later, they accept lots of medical symptomatology as being a normal part of life.” |
| **Normal functioning family life disrupted** | | | |
| Civil society | | | “In South Africa, the makeup of the family is so different, particularly in our townships and in our communities. We have from child headed households to households managed by the granny or the gogo [grandmother], single mom households. […] So we've had many times where families, the mommy’s here, […] and the young adult son or daughter must take care of the younger siblings.” |
| Civil society | | | “[…] the requirements of treatment and having a family back at home where they have their husband or their spouse or partner at home and more children at home and not being able to provide financially to the home.” |
| Healthcare professional | | | “It's a cost emotionally on the patient, they’re kids, they have to come with the parents. Now where's the parents staying, what’s the parent eating? Then they miss out on school, because again, they have to be admitted.” |
| **Adherence and defaulting** | | | |
| **Defaulting on treatment** | | | |
| Healthcare professional | | | “And then it's an education problem, often. You know, the parents agree to something but the family are completely against it. And then patients are lost to follow-up, they don't come back, especially when we're talking about big surgeries, like amputations and inoculations and just the general resistance to surgery.” |
| Healthcare professional | | | “If you do start the treatment, they do see a response to the treatment, they think that the child is cured, and they default follow-up. And that's one of the reasons why they default follow-up.” |
| Civil society | | | “Because that has happened often, where parents default on treatment because accessibility and finances is a challenge.” |
| **Multisectoral support to prevent defaulting** | | | |
| Civil society | | | “But CHOC [Childhood Cancer Foundation] has made a very, very big impact in terms of helping parents to access the pediatric oncology wards. So the defaulting statistics have definitely decreased. And because also not only the transport fund, but the role of the social worker in the ward.” |

| **MONITORING AND SURVEILLANCE** | |
| --- | --- |
| **Lack of monitoring and surveillance** | |
| Policy maker/ regulator | “So in the Western Cape, they're building an electronic health record. You can see somebody was seen at a clinic. Somebody could look at this from the outside and go, hang on that doesn’t look right. In a rural area, that paper record is inaccessible. And we don't know who's being missed, nobody is checking.” |
| Policy maker/ regulator | “And we've got a problem with our cancer registry, our cancer reporting system, that is underfunded, not terribly accurate, and hampers our ability to use those data to advocate.” |

| **CROSS-CUTTING EMERGING THEMES** | |
| --- | --- |
| **Advocacy** | |
| **Need for advocacy** | |
| Policy maker/ regulator | “But I think it also needs a lot of advocacy from various role players to try encourage people to… or companies to produce these medications.” |
| Medicine supplier | “I would say the health activists to get involved, is to make sure that the important childhood cancers are picked up and reflected under the prescribed minimum benefits.” |
| Policy maker/ regulator | “So what I like to say is that I don't hear anything around patient access for pediatric oncology. And one would expect somebody in my position and my traverse in the environment that I would hear that, that somebody would be shouting at me, somebody would be criticizing us, somebody would be saying something, and I'm hearing nothing of that.” |
| **Awareness** | |
| **Lack of awareness on childhood cancers** | |
| Healthcare professional | “I think awareness and education is probably the most important and when we talk about it, we’re talking about awareness and education among healthcare workers, primary health care workers, firstly, and then go a step further and say, even traditional healers, and then going even further and saying in the general population.” |
| **Lack of awareness on health system components** | |
| Medicine supplier | “And then that referral pathway is not clearly defined. People don't know how to go through the system, or how to navigate the system.” |
| Policy maker/ regulator | “But also in the private sector, […] what medical aid schemes do is they will publish their medical aid scheme rules for example, then you would get members that don't read. What they are eligible for, what they can access and what not. So I see that is a major problem as well.” |
| **Equity** | |
| **Inequities in care and services provided** | |
| Policy maker/ regulator | “[…] the differences in per capita allocation between private and public dramatically differ. So just the resources available for a child in the private sector are very different from resources available in the public sector.” |
| Policy maker/ regulator | “Because if you are in a rural area, you can’t have access to a specialist. They might miss the diagnosis. But if you are in a city where there's access to tertiary hospitals, whether it’s the private or public sector, you've got a bigger chance of your condition being diagnosed early.” |
| Healthcare professional | “Why should there be a difference between what I get in the Northern Cape versus what I get in the Free State versus what I get in the Western Cape? It all comes from the same taxpayer” |
| **Non-governmental organizations (NGOs)** | |
| **Services and support provided in NGO sector** | |
| Civil society | “At this stage, because of the lack of services being provided by government, or in the private sector, it is the nonprofits that are providing the services. And that would be from early detection, or creating awareness, right through to psychosocial support, right through to end of life care. So if it hadn't been for the nonprofits, we would have certainly a disaster in this country.” |
| Healthcare professional | “But we do depend quite heavily on NGOs, for assistance with a lot of things, for housing, for outpatients, for transport money, even to hand out food packets when they come just as an incentive for them to come to the hospital.” |
| **Lack of funding for NGOs** | |
| Civil society | “I think one of the things that is a challenge is the fact that CHOC [Childhood Cancer Foundation] does not receive any government funding. And yet we provide such a crucial support role to the to the state's patients, you know, and I think that more assistance, financial assistance is needed from the state to charities in the cancer sector.” |
